# Use of Mass Spectrometry to Screen Glycan Early Markers in Hepatocellular Carcinoma

**DOI:** 10.3389/fonc.2017.00328

**Published:** 2018-01-15

**Authors:** Raphaela Menezes de Oliveira, Carlos Andre Ornelas Ricart, Aline Maria Araujo Martins

**Affiliations:** ^1^Laboratory of Biochemistry and Protein Chemistry, Department of Cell Biology, Institute of Biological Sciences, University of Brasilia, Brasilia, Brazil; ^2^University Hospital Walter Cantídeo, Surgery Department, Federal University of Ceara, Fortaleza, Brazil

**Keywords:** glycans, hepatocellular carcinoma, mass spectrometry, glycomics, biomarkers, cancer

## Abstract

Association between altered glycosylation patterns and poor prognosis in cancer points glycans as potential specific tumor markers. Most proteins are glycosylated and functionally arranged on cell surface and extracellular matrix, mediating interactions and cellular signaling. Thereby, aberrant glycans may be considered a pathological phenotype at least as important as changes in protein expression for cancer and other complex diseases. As most serum glycoproteins have hepatic origin, liver disease phenotypes, such as hepatocellular carcinoma (HCC), may present altered glycan profile and display important modifications. One of the prominent obstacles in HCC is the diagnostic in advanced stages when patients have several liver dysfunctions, limiting treatment options and life expectancy. The characterization of glycomic profiles in pathological conditions by means of mass spectrometry (MS) may lead to the discovery of early diagnostic markers using non-invasive approaches. MS is a powerful analytical technique capable of elucidating many glycobiological issues and overcome limitations of the serological markers currently applied in clinical practice. Therefore, MS-based glycomics of tumor biomarkers is a promising tool to increase early detection and monitoring of disease.

## Introduction

The definition of glycans includes any form of mono-, oligo-, or polysaccharides free or disposed in glycoconjugates, which can be divided into glycoproteins, glycolipids, and glycosaminoglycans (GAGs) (1, 2). Some of the first studies of glycans, such as specific tumor markers, notice that poor prognostic in different cancers, such as breast and colon carcinoma, were related to aberrant glycosylation in glycoproteins (3, 4).

The role of glycans in the progression of cancer was initially proposed with the emergence of monoclonal antibodies against oncofetal carbohydrate antigens present in many cancers (5, 6). Since then, glycans from both neoplastic and regular cells were recognized as mediators of many pathophysiological events in cancer progression (3, 4, 7–9). For instance, activation of oncogenes and loss of tumor suppressors cause glycosylation alterations in neoplastic cells (7, 10). In tumor environment, changes such as fucosylation, sialylation, and high-mannose structures, provide increased proliferative capability, enabling the tumor cells to control key events for malignancy development. The most aggressive set of these changes include modulation of cell adhesion systems and cell motility, resulting in a “Darwinian selection” which promotes metabolism imbalance, cell invasion, and subsequent metastasis (6, 9, 11, 12). The migrating tumor cells can interact with leukocytes, endothelial cells, and platelets of lymphatic or blood vasculature through a unique highly expressed or altered glycan, such as Lewis blood antigens and *N*-acetyllactosamine, thus amplifying the metastatic spread (13–15).

According to estimation, glycosylation is present in more than 50% of human proteins (16, 17). These glycoproteins are synthesized in various glycoforms which differ in placement site and molecular glycan structure. Concerning glycoproteins, the initial attachment of carbohydrate determines two main conjugation forms: *N*-glycosylation, when is linked to the amino group of asparagine, and O-glycosylation, when is linked to hydroxyl group of serine or threonine (18).

Most glycoproteins are functionally arranged on cell surface and extracellular matrix (ECM) favoring mediation of cell interactions and signaling (6, 9). However, a matrix with few glycans favors invasion and metastatic potential in a wide range of tumors (9, 14). In 2009, Gornik and collaborators concluded that environmental changes and pathological development results in alterations in carbohydrate profile, revealing diagnostic potential (19).

Since posttranslational modifications (PTM) are important regulators of molecular machinery, altered phenotype resulting from glycan biosynthesis in pathological conditions may be more responsive than those related to changes in protein expression. Moreover, glycan modification combined with protein expression profiling can optimize the monitoring of development and progression of malignancies.

In 2010, a publication of Lauc and collaborators suggested that glycosylation status could indicate different risk factors for diseases (20). In fact, some tumor biomarkers presently used in clinical practice are glycoproteins found in blood, such as CEA, CA19-9, and CA-125 for colon, pancreatic, and ovarian cancer, respectively (21–24).

Several studies were conducted to clarify the molecular events that result in liver tumor phenotypes, especially those linked to the emergence of hepatocellular carcinoma (HCC) (25–31). The late detection through biochemical tests and/or image diagnosis, generally in advanced stages of the disease, makes the HCC, one of the most aggressive cancers in the world (32, 33). This scenario shows the need for detection and validation of new biomarkers, highlighting the importance of glycomics approach, in characterization and control of glycosylation to establish the overall molecular profile of the pathological development process.

## Importance of Glycans in Cancer

The initial perception of glycans selection expressed on tumor cells resulted from their capacity to bind to a variety of plant lectins (34). Despite the diversity of glycoforms, restricted sorts of glycan structures are related to progression and poor prognosis in cancer (12).

Most cell surface receptors are *N*-linked glycoproteins, such as transforming growth factor receptor beta, epidermal growth factor receptor (EGFR), and integrins (35). Many secreted signaling molecules, such as growth factors and hormones, interact with cells *via* glycan-lectin interaction, often forming galectins and siglecs complexes (36, 37), which increase receptor availability on the cell surface (36, 38). Endogenous lectins enable essential processes such as quality control of secreted proteins, cell interactions, adhesion, and motility; therefore, the interaction of *N*-glycans in glycoproteins with these molecules is critical for cellular adaptation during cancer (9, 39).

Another aspect of glycosylation in cancer is the upregulation of terminal glycan structures, such as tetrasaccharide NeuNAcα2,3Galβ1,4(Fucα1,3)GlcNAc, also known as sialyl Lewis^x^ (sLe^x^) (3, 7). The sLe^x^ structures are mainly detected in β1-6GlcNAc branches carrying *N*-acetyllactosamine, the preferred ligand for galectin-3 (40). Increase of branching β1,6GlcNAc *N*-glycans is related to metastatic behavior of tumor by changes in growth factor receptors on the surface of tumor cells (41, 42). The sLe^x^ antigen was associated with progressive metastatic behavior and consequent poor prognosis due to its function in neoplastic cells adhesion to endothelium (12, 15, 43). The antigen present discretely in vascular glycoproteins is the main binder of selectins and through this interaction, are able to mediate the early steps of leukocyte adhesion to endothelium (3, 35, 44). Furthermore, hematogenous spread is the usual route of migration in carcinomas; thus, tumor cells carrying sLe^x^ in the bloodstream can be linked to vascular selectins and contribute to cancer progression (15, 43, 45–47).

Presentation of sLe^x^ antigen and truncated forms of O-glycans were widely observed changes in cell carcinomas (34, 47, 48). This type of cancer, in particular, expresses modified glycans on mucins, enabling binding to a variety of receptors and the consequent modulation of the biological properties of tumor cells (10, 14). Many mucins, such as MUC1, MUC2, and MUC4, are associated with tumor progression (24, 49, 50).

Mucins are glycoproteins of epithelial cells heavily O-glycosylated that may comprise up to 80% by weight of the molecule (6). Although O-glycosylation is the regular classical pathway, N-glycosylation of mucins can also occur, either in normal or modified conditions (16, 51, 52). The common structural characteristic of mucins is a tandem domain rich in serine, threonine, and proline residues (24). The first step in O-glycosylation is the linkage of *N*-acetylgalactosamine to these amino acid residues, also termed Tn antigens (12, 53).

Impairment of the glycosylation process of mucins, or by dysregulation expression of the mucin gene or enzyme involved in the biosynthesis of O-glycan, produces a high frequency of shorter structures, such as Tn antigens, T antigens, and both sialylated forms (50, 54).

The high expression of early Tn chains terminated by sialylation, the sialyl-Tn, reduces cellular interactions and increases the metastatic potential (18, 55, 56). In other words, such molecules confer ability to travel through the ECM and to detach from the primary tumor (14). The sialylation of T antigens found in carcinomas is a consequence of increased expression of sialyltransferases, enzymes responsible for transfer sialic acid to glycoforms (57).

Glycosaminoglycans is a family composed by linear polysaccharides, mostly sulfated, containing repeating disaccharide units and present mainly in cell surfaces and extracellular matrices (9, 58). Through interaction with proteins, GAGs regulate several physiological processes acting mainly as “co-receptor,” facilitating the formation of receptor–ligand complexes and reducing the effective concentration of ligand required for receptor activation (9, 58). Moreover, they favor the hoarding of binders for upcoming mobilization and shield against degradation (6, 59). In the tumor microenvironment, such functions are destitute, enabling growth factors released by the tumor exert autocrine and paracrine effects on surrounding host cells, such as endothelial cells (9).

Altered microenvironment is known to be involved in all stages of malignant progression, from initial transformation to invasion and metastasis, and many pathologists have recognized that some tumors are heavily infiltrated by both innate and adaptive immune system cells, thereby promoting inflammatory conditions in non-neoplastic tissue (32, 60, 61). The interaction between accessory cells, their mediators, neoplastic cells, and structural components of the ECM, such as glycans, regulate all aspects of tumorigenicity (60).

## Importance of Glycomics Approach in Early Markers Discovery

By analogy with genomics, transcriptomics, and proteomics, the glycomics refers to studies that seek to define and quantify glycans in a cell, tissue, or organism (1, 6).

The challenge of glycomics is enormous, whereas glycosylation of proteins and lipids correspond to one of the most abundant PTM and also more structurally diverse (1, 62–64). Glycans often affect the functionality of the carrier proteins, so the joint analysis of these molecules, defined as glycoproteomics, is essential to understanding the universal role of glycans (7). However, the characterization of glycans in serum glycoproteins still is a challenge owing to the variety of glycoforms (10, 62, 65).

There are only about 20 biomarkers approved and used clinically by the Food and Drug Administration (FDA), of which just 5 are validated for application in diagnosis (66, 67). Many of these biomarkers do not have sufficient specificity and sensitivity, and this may be explained by the readout based only in protein levels (12, 67). Protein abundance may not correlate with the stage of the disease, may differ among individuals and float in other diseases, especially those with inflammatory origin (12, 68).

In glycomics and glycoproteomics approach, comparison of overall glycan profiles in tissues or body fluids of interest allows the recognition and utilization of a new category of tumor biomarkers (1, 62, 69). An ideal tumor biomarker would allow a simple blood test that can aid in clinical decisions to evaluate the risk of developing a disease, to permit early diagnosis and to characterize the disease stage, as well to monitor disease progression and treatment efficacy (10, 12).

Through advances in physical and biological chemistry, the field of glycobiology acquired the tools necessary to elucidate the composition and structure of glycans associated with cells and proteins (65). The glycomics study of tumor biomarkers and drug targets became an attractive field of research and widely exploited, as the importance of glycosylation alterations emerged (2, 18).

## Importance of HCC Glycans Early Markers

Hepatocellular carcinoma is the most important late complication of chronic degenerative processes of the liver, derived from a primary liver disease (18, 32, 70–72). In 2012, about 14.1 million of new cancer cases were registered, including 5.6% corresponded to liver tumors, which is highlighted as within the five most common types of cancer in men (73).

The risk factors of HCC occurrence are well defined in the literature nowadays and among them, cirrhosis plays a major important role, along with hepatitis B virus and hepatitis C virus infection (32, 72, 74). Alcohol abuse and metabolic syndromes, such as diabetes, also contributes as relevant risk factors (32, 72).

In current clinical practice, biopsy and diagnostic imaging, recommended by Barcelona and Milan criteria are the most reliable methods for detection of liver diseases (10). However, liver biopsy is not a recommended procedure for patients with HCC because it could allow physical tumor metastasis and imaging investigation is not able to reveal tumors in primary stages. Thus, there is a great urgency to use non-invasive markers that can routinely assess the progression of HCC and other liver diseases (10, 23, 32, 62, 75).

The prognosis of patients with HCC presents rate of only 18% survival at 5 years, partly attributed to the diagnosis in advanced stages of the disease (76). One of the last curative resources in advanced HCC is orthotopic transplantation of the organ, whose recurrence reaches 10–20% of receptors, despite the use of strict criteria in the selection of candidates (77). The conventional system of tumor–node–metastasis staging is flawed in precognition of tumor recurrence after surgery owing to remarkable heterogeneity among patients at the same stage of HCC (78). Molecular approaches to stratify HCC patients through biomarker integration can assist in patient survival prediction and improve their prognostic stratification (79).

Most serum glycoproteins are from hepatic origin, so it is inferred that liver diseases associated with abnormal glycosylation can display significant changes in glycoproteins (10, 18, 69). Previous works demonstrated that different phenotypes such as liver fibrosis, cirrhosis, and HCC features may be unravel by such changes in serum glycoproteins (71, 80–84).

Increased alpha-fetoprotein (AFP) is usually resulting from liver disease and changes at its glycoform is a good indicator of disease progression (85, 86). Many physiological conditions may affect serum levels of AFP, restricting the specificity of the marker, and the low detection sensitivity often contributes to recognition of HCC already in advanced stages (29, 80). The establishment of biomarkers of early expression can mitigate these difficulties and assist in differential diagnosis of liver diseases (80). In 2006, the glycosylated fraction AFP-L3, was approved by the FDA as an additional marker in early detection of HCC, since the sole determination of AFP concentration was not able to reveal initial stages of disease (87).

Studies correlating glycosylation patterns with HCC measure either activity or regulation of specific glycosyltransferases. However, in most cases, it was not found correlation between regulation of those enzymes and structural changes at the cell surface glycans (18).

Altered mannose structures have been reported frequently in the literature in several cancers, including HCC (88–90). Studies comparing purified carbohydrate chains from HCC against liver tissue notice that chains with high-mannose levels were found exclusively on pathological conditions (90, 91). The abundance of these molecules may facilitate not only the proliferation, adhesion, and tumor metastasis but also interactions with specific endothelial cells, determining in which organ or tissue is most likely the secondary tumor site (88). This fact reinforces the role played by microenvironment in metastasis formation first postulate by “soil-seed theory” of Stephen Paget (92).

Heparan sulfate (HSPG) is a sort of GAG, commonly reported in proteoglycans, associated with tumor cell proliferation (9). In HCC, as well as pancreas, breast, and ovary cancers, tumor cells are able to regulate Hsulf-1 gene and consequently modulate sulfation of cell surface HSPGs in such way that promotes increased ability to bind to growth factors and better activation of tyrosine kinases receptors (93).

**Figure 1:**
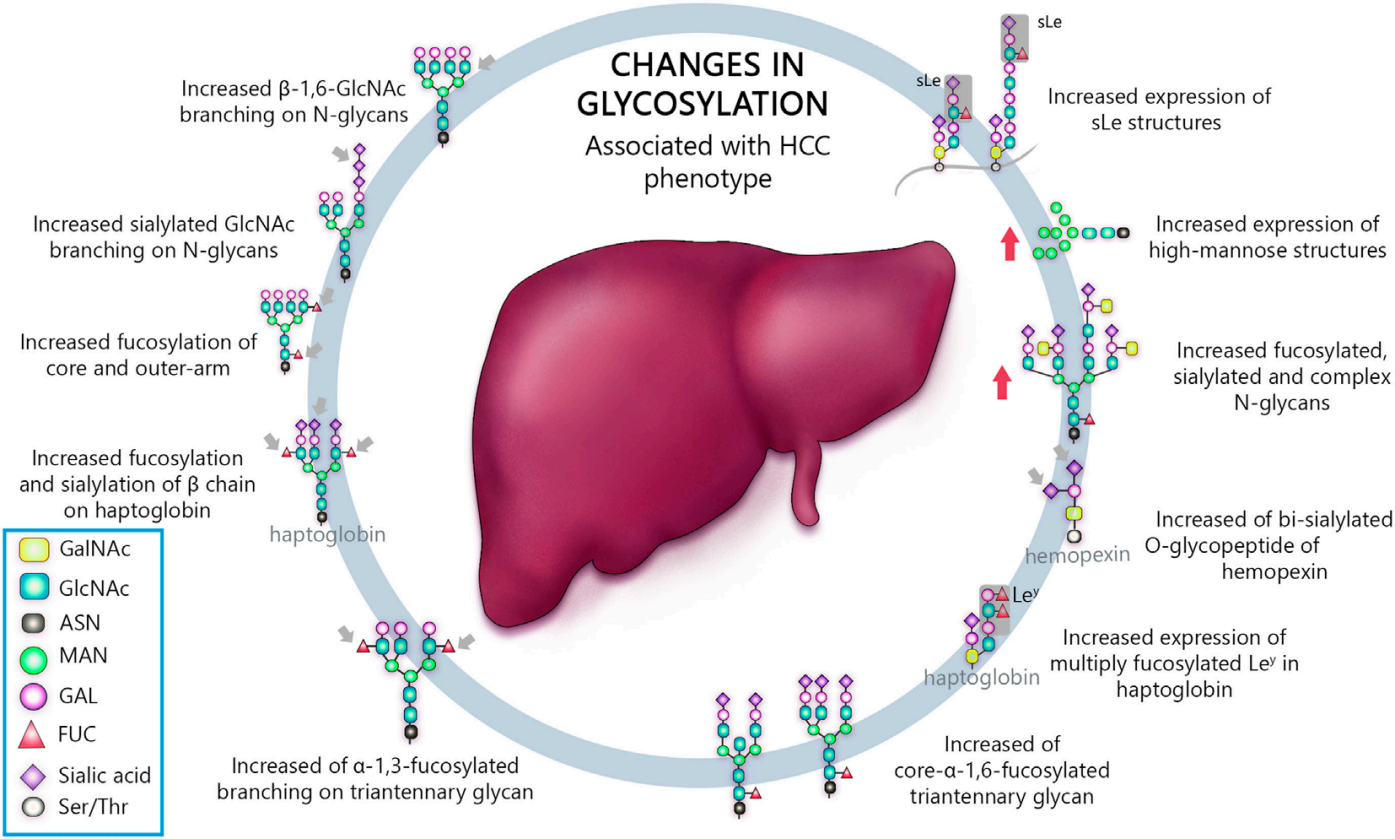
Schematic representation of the most important glycan alterations associated with hepatocellular carcinoma (HCC) phenotype.

Venetz and collaborators correlated *N*-glycan structures and cancer-specific antibody effectiveness, and reported that non-sialylated glycans have a negative impact on the establishment of disease (8). Chia and collaborators suggested that the elucidation of short O-glycans structures regulation would expand therapeutic perspective, since many attempts to use such molecules as biomarkers, antibody, and therapeutic vaccine have failed, due to low sensitivity and/or specificity (12). Liu and collaborators evaluating HCC patients who underwent hepatectomy established correlation between high expression of β1,6-*N*-acetylglucosaminiltransferase V (Mgat5) and recurrence and poor survival after procedure, revealing potential as an unfavorable biomarker (26). Finally, several works, summarized in Table 1, have shown an association between aberrant glycosylation profile and HCC phenotype (25, 27, 29, 47, 80, 90, 93–105). Thus, the most important types of glycosylation are show in Figure 1.

**Table 1:** Changes in glycosylation associated with hepatocellular carcinoma phenotype.

Glycan modification	Proposed major function	Reference
Increased β-1,6-GlcNAc branching on *N*-glycans	Decreased cell adhesion to fibronectin	(110)
Increased sialylated GlcNAc branching on *N*-glycans	Decreased cell–cell interaction, enhanced motility and invasiveness	(110)
Increased fucosylation of core and outer-arm	Decreased cell polarity and altered adhesive properties	(99–102)
Increased fucosylation and sialylation of β chain on haptoglobin	Altered adhesive properties	(83)
Increased expression of sLe structures	Enhanced tumor cell adhesion during metastasis	(25, 47)
Increased expression of high-mannose structures	Enhanced proliferation, adhesion, and metastasis	(25, 29, 95)
Increased fucosylated, sialylated, and complex *N*-glycans	Increase activity of the epidermal growth factor receptor (EGF-R), decreased cell–cell interaction, enhanced motility and invasiveness	(27, 103, 105–107)
Increased of bi-sialylated O-glycopeptide of hemopexin	Altered adhesive properties	(107)
Increased expression of multiply fucosylated Le^y^ in haptoglobin	Enhanced tumor cell adhesion during metastasis	(108)
Increased of core-α-1,6-fucosylated triantennary glycan	Enhanced cell migration	(109)
Increased of α-1,3-fucosylated branching on triantennary glycan	Decreased cell polarity and altered adhesive properties	(104)

The techniques for surveillance of HCC remains controversial. Although suggestions that biomarkers improve early detection, the usual serum molecular markers that are used, such as AFP, des-γ-carboxyprothrombin (DCP), and the L3 fraction of AFP (AFP-L3), are all more frequently associated with advanced stage disease and there is no evidence that leads to improved cure rates (32). The use of glycans as markers is a considerable alternative and also a challenge for the diagnosis of diseases, since biochemical environment alterations could impact in glycosylation machinery (69).

## Mass Spectrometry (MS) as a Tool for Glycomics Analysis

**Figure 2:**
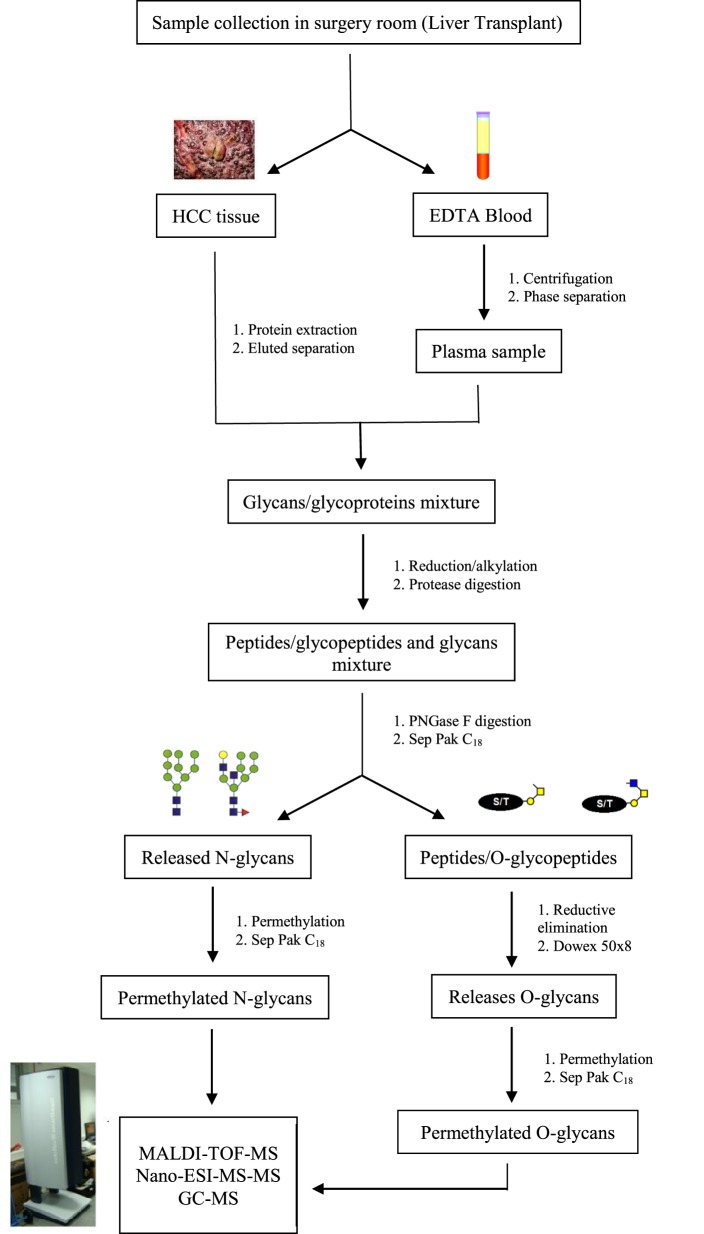
Schematic representation of the preparation process for glycomics analysis in mass spectrometry biomarkers research. Protocols for glycoprotein analysis depend on the amount of sample. Since biomarkers research often use tissue or serum/plasma samples, the strategy adopted does not require presence of SDS for protein denaturation but is only applicable if more than 50 mg of protein is available. If you are not interested studding O-glycosylation in the moment, you can storage the peptides/O-glycopeptides and proceed directly to permethylation of *N*-glycans.

In recent years, challenges in glycomics have been unraveled by many innovative approaches and methods, accelerating the acquisition of glycobiological data. While investigation methods and protocols for glycomic and glycoproteomic studies progressed, glycobiological challenges have been reduced and the effects of specific glycans gradually determined. Among these methods, MS showed to be an essential tool for the expansion of glycosylation biological meaning (105). Figure 2 demonstrates a protocol frequently used for glycomic analysis in MS biomarkers research.

Complex mixtures of glycans, derived from clinical samples for example, are often separated by chromatography or capillary electrophoresis and detected by fluorescence or MS. Fluorescence detection requires fluorescently tagged glycans and results in partial structural data from chromatographic or electrophoretic retention times, while MS can also be applied on native oligosaccharides and may provide detailed structural data through increasement of resolution power, such as Fourier transform ion cyclotron resonance or techniques such as hydrogen deuterium exchange mass spectrometry and ion mobility separation (IMS) (1, 28, 102, 106). MS can clarify glycan structural characteristics that are difficult to elucidate without deglycosylation and, if combined with glycopeptide analysis, provide a thorough knowledge of the micro and macro heterogeneity of protein glycosylation (107).

A promising non-invasive strategy to discover early biomarkers of cancer is characterizing glycomic patterns in pathological serum or plasma and MS, in particular, is an enabled technology for this analysis (105, 108). The constant advancement rendered the N- and O-glycomic characterization possible nonetheless there is no consensus on the ideal method of investigation and each one presents both advantages and disadvantages. Specifically, for concomitant examination of both *N*- and O-glycan structures, porous graphitized carbon separation with electrospray ionization (ESI) followed by MS/MS has been highlighted as a successful workflow (1).

Matrix-assisted laser desorption/ionization mass spectrometry (MALDI-MS) is an effective technique for N-glycomic analysis of clinical samples and has been widely applied to identify *N*-glycans in HCC (27, 109). Its relatively tolerance to salt and other contaminants permit simple sample preparation after *N*-glycan enrichment. Kim and collaborators evaluated the performance of a quantitative MALDI targeting glycomics approach, in the analysis of *N*-glycans, in both, purified AFP from normal cord blood (control condition) and from HCC cell line—Huh7 cells—(increase of core-fucosylation). It was demonstrated that the results are promising in clinical and bioengineering fields (110). In 2009, Lattová and collaborators examined serum of animal models with HCC-inducted through MALDI-MS, identifying forty different *N*-glycans, in which some structures appeared to have highest frequency in HCC samples (29). Kamiyama and collaborators, also applying MALDI-MS analysis, reporting two *N*-glycan structures, G2890 and G3560, which were presented as potential biomarkers of malignancy in HCC (27).

Another pragmatic technique for investigation of glycans and glycoproteins in body fluids is the ESI-MS, therefore, potentially cancer biomarkers can be assigned by its use and in HCC field, this is already widely exploited (105, 111). In 2013, Zhang and collaborators through LC–ESI-MS/MS analysis reported that β haptoglobin chain sialylated and fucosylated glycoforms may be associated with early hepatocarcinogenesis therefore may be useful as differential monitoring markers for patients with cirrhosis and HCC (80). Another research group also demonstrated with LC–ESI-MS that fucosylated haptoglobin structures are upregulated in HCC if compared to cirrhotic samples (112). Tsai and collaborators also through LC–ESI-MS approach, and comparing serum samples from HCC and cirrhotic patients, inferred 11 *N*-glycan structures with different regulation between the 2 biological conditions and therefore possible biomarkers candidates (105). As notice, the specificity of these MS methods, MALDI-MS and ESI-MS, is frequently related to the raw intensities of glycan ions in the two contrasting scenarios, non-pathological versus pathological samples (28).

During recent years, to include information regarding the number and abundance of distinct isomers and conformers, separation methods, such as liquid chromatography (LC) and IMS, has been frequently associated to MS. ESI is especially well suited to LC–MS approach and according to Hu and Mechref, the system formed possibly presents higher sensitivity than MALDI-MS and LC−MALDI-MS in detecting permethylated *N*-glycans from serum (111). Isailovic et al., analyzing *N*-glycans to describe diseases states, combined IMS-MS and a three-dimensional IMS-IMS-MS technique that aims to describe variations in structure based on creation of new conformations instead of ion fragments (28). Statistical analyses of these IMS distributions proposed that deviations in isomer distributions may denote some disease states, showing that IMS is suitable to complement MS analysis.

Mass spectrometry-based glycomic and glycoproteomic investigation require, besides the appropriate equipment, a fair amount of expertise. Glycoproteomic approaches usually consist of numerous steps but, in recent years, alternative strategies for production of site-specific structural insights of *N*- and O-glycans are being optimized (113, 114). Whole glycopeptide analysis using a blend of distinct MS fragmentation techniques or collision energies that fragment peptides or glycans, awakens the possibility of discovering new complex diseases markers; therefore, it may be of great value for the study of glycan early markers in cancer (1, 113, 114).

An additional novel and promising MS approach for detection of *in situ* glycan alteration of specific proteins is mass spectrometry imaging (MSI) (115). This method produces for each ionization point a spectrum that presents structural data that may be correlated with the analyte spatial distribution in targeted tissue. MSI has been improved for glycomic studies and can generate N-glycosylation signatures of a variety of tissues (116), including formalin-fixed paraffin-embedded tissues (117). When applied on HCC tissues, MALDI imaging was able to spatially identify *N*-glycan compositions and distingue global profiling from non-pathological tissues (118).

Recently, real-time *ex vivo* MS for tumor detection has been performed as an alternative for histological diagnose. In general, the standard equipment and sample preparation, essential to produce MS data, is very elaborate for clinical use. Since rapid evaporative ionization mass spectrometry was apply to a surgical instrument (iKnife) in order to recognize lipid profiling of regular and cancerous human breast tissue, other adaptations in operating room have also simplified the physical structure and requirement for specialists, supporting the clinical purpose (119, 120).

Although MS is the most suitable technique for analysis of complex glycan samples, glycomics is currently insidious due to structural characterization and automation, to become a regular choice of investigation in clinical area. However, analysis of other biomolecules (such as lipids and metabolites) have already been implemented as cutting-edge MS application, not only in clinical practice but also in surgical center, such as MasSpec Pen technology (120–123).

## Conclusion

Glycans are involved in numerous essential biological functions that are altered in tumor and tumor microenvironment, suggesting that such components are intimately associated with the neoplastic condition. Due to its biopathological characteristics and lack of an early biomarker, HCC display very limited curative options. HCC patients usually present late diagnosis detection, when the cancer is already in advanced stages. The absence of specificity in the laboratory diagnosis, inconclusive image analysis, and invasive characteristic of liver biopsy procedure contribute to the poor prognosis of these patients. A clear view on initial clinical stages of the disease is a determining factor for choice of curative treatment, as well as for its success. Non-invasive markers not only measure the risk of developing HCC and other liver diseases in its early stages, as it can routinely evaluate its progression. Current advances in glycobiology field could help vanquish the several issues remain to be understood, particularly in complex diseases such as HCC. MS proved to be an enabling tool for non-invasive strategies in future validation of potential glycobiomarkers and translation in clinical venue. In this scenario, the study of glycans and its characteristics represents a hopeful implement for better knowledge of cancer biological properties, especially in HCC. The emerging concept of translational medicine has drawn a lot of attention in the last decade and one of the premises is the implementation of high-performance techniques in clinical settings to demystify the usage of analytical tools in downstream services that might have high impact on patients’ health.

## Author Contributions

RO has contributed to the drafting, writing, and revision of the manuscript. CR has contributed to writing and revision of the manuscript. AM has contributed to the conception, writing, and revision of the manuscript. All authors approved the review final form.

## Conflict of Interest Statement

The authors declare that the research was conducted in the absence of any commercial or financial relationships that could be construed as a potential conflict of interest.
